# Pharmacokinetic equivalence of CT‐P39 and reference omalizumab in healthy individuals: A randomised, double‐blind, parallel‐group, Phase 1 trial

**DOI:** 10.1002/clt2.12204

**Published:** 2022-11-15

**Authors:** Marcus Maurer, Sarbjit S. Saini, Kristi McLendon, Paul Wabnitz, Sunghyun Kim, Keumyoung Ahn, Suyoung Kim, Sewon Lee, Clive Grattan

**Affiliations:** ^1^ Institute of Allergology Charité – Universitätsmedizin Berlin Corporate Member of Freie Universität Berlin and Humboldt‐Universität zu Berlin Berlin Germany; ^2^ Allergology and Immunology Fraunhofer Institute for Translational Medicine and Pharmacology (ITMP) Berlin Germany; ^3^ Johns Hopkins University School of Medicine Johns Hopkins University Baltimore Maryland USA; ^4^ Nucleus Network Pty Ltd Herston Queensland Australia; ^5^ cPHARMA Pty Ltd Adelaide South Australia Australia; ^6^ Celltrion, Inc. Incheon Republic of Korea; ^7^ St John's Institute of Dermatology Guy's Hospital London UK

**Keywords:** biosimilar, CT‐P39, immunoglobulin E, omalizumab, pharmacokinetics

## Abstract

**Background:**

CT‐P39 is being developed as a biosimilar of reference omalizumab. This study aimed to assess the pharmacokinetic equivalence of CT‐P39 to European Union‐approved and United States‐licensed reference omalizumab (EU‐ and US‐omalizumab, respectively).

**Methods:**

This two‐part, randomised, parallel‐group, double‐blind Phase 1 trial (NCT04018313) was conducted in healthy individuals with a total immunoglobulin E (IgE) level ≤100  international units (IU)/ml at screening. In part 2, described herein, participants were randomised (1:1:1) to receive a single 150 mg subcutaneous dose of CT‐P39, EU‐omalizumab, or US‐omalizumab. The primary endpoint was pharmacokinetic equivalence in terms of area under the concentration–time curve (AUC) from time zero to the last quantifiable concentration (AUC_0–last_), AUC from time zero to infinity (AUC_0‐inf_), and maximum serum concentration (*C*_max_). Equivalence was concluded if 90% confidence intervals (CIs) of the geometric least‐squares means ratios were contained within the predefined 80%–125% equivalence margin. Additional pharmacokinetic parameters, pharmacodynamics, safety, and immunogenicity were also evaluated.

**Results:**

Overall, 146 participants were randomised (CT‐P39, *N* = 47; EU‐omalizumab, *N* = 49; US‐omalizumab, *N* = 50). For all primary pharmacokinetic parameters, 90% CIs for pairwise treatment comparisons were within the 80%–125% equivalence margin, demonstrating pharmacokinetic equivalence. Decreases in free IgE and increases in total IgE serum concentrations were comparable across groups. CT‐P39 was well tolerated. Safety endpoints were comparable across groups: there were no treatment‐related serious adverse events, deaths, or discontinuations due to treatment‐emergent adverse events.

**Conclusions:**

CT‐P39 was well tolerated and demonstrated pharmacokinetic equivalence with EU‐omalizumab and US‐omalizumab following administration of a single dose in healthy individuals.

## 1 INTRODUCTION

Omalizumab is a humanised monoclonal antibody targeting immunoglobulin E (IgE).^1^ IgE initiates an allergic cascade when it binds to the high‐affinity IgE receptor (FcεRI) on mast cells and basophils; omalizumab binding reduces the amount of free IgE, leading to FcεRI receptor downregulation and inhibition of IgE‐mediated inflammation.^2^, ^3^ Reference omalizumab (Xolair^®^; Novartis) received initial approval from the US Food and Drug Administration (FDA) in 2003 and the European Commission (EC) in 2005.^2^, ^3^ FDA approval has been received for indications comprising asthma in adults and paediatric patients aged ≥6 years, nasal polyps in adults, and chronic spontaneous urticaria in adults and adolescents aged ≥12 years^3^; EC approval has been received for allergic asthma in patients aged ≥6 years, chronic rhinosinusitis with nasal polyps in adults, and chronic spontaneous urticaria in adults and adolescents aged ≥12 years.^2^ Accordingly, global, European, and US treatment guidelines recommend the use of omalizumab in selected patients as an add‐on therapy for uncontrolled moderate or severe asthma,^4^, ^5^, ^6^, ^7^ which may include allergic and non‐allergic eosinophilic asthma,^5^ in the treatment of nasal polyps,^8^ and as add‐on therapy for chronic urticaria refractory to a high‐dose, second‐generation H_1_‐antihistamine.^9^, ^10^, ^11^

The cost of biologic therapies used to treat allergic diseases is high, which can limit patient access to these treatments.^12^, ^13^, ^14^, ^15^ Biosimilars can offer cost savings, potentially allowing more patients globally to benefit from biologic therapy,^14^, ^15^ and the future potential role of biosimilars of reference omalizumab has been mentioned in British treatment guidelines.^11^ Biosimilars are assessed via dedicated FDA and European Medicines Agency pathways, and regulatory approval requires demonstration that there are no clinically meaningful differences in terms of safety, quality, and efficacy between the candidate biosimilar and original biologic/reference product.^16^, ^17^ Ultimately, regulatory approval is based on the totality of evidence gathered through a stepwise comparability exercise involving analytical, non‐clinical, and clinical studies, which together evaluate pharmacokinetics (PK), efficacy, safety, immunogenicity, and potentially pharmacodynamics (PD), of the candidate biosimilar.^16^, ^17^, ^18^

No biosimilars of reference omalizumab have yet obtained regulatory approval from the FDA or EC.^19^, ^20^ However, CT‐P39 is in development as a reference omalizumab biosimilar, and as part of the development programme, a two‐part Phase 1, single‐dose, double‐blind, parallel‐group, randomised study was conducted. Part 1 was a first‐in‐human study to evaluate the initial safety of CT‐P39 versus European Union‐approved reference omalizumab (EU‐omalizumab): no safety issues were identified and CT‐P39 was well tolerated (unpublished data on file). Part 2, described in this article, aimed to demonstrate the PK equivalence of CT‐P39 to EU‐omalizumab and United States‐licensed reference omalizumab (US‐omalizumab), all administered via prefilled syringe (PFS). PD, safety, and immunogenicity were also evaluated. Two comparators were used to satisfy US and EU regulatory requirements to demonstrate similarity to a reference product marketed in the respective regions.

## 2 METHODS

### 2.1 Study design

This was a two‐part, randomised, parallel‐group, double‐blind, Phase 1 trial (ClinicalTrials.gov: NCT04018313) conducted in healthy individuals at two clinical research centres in Australia (Nucleus Network Pty Ltd and CMAX Clinical Research Pty Ltd). A parallel‐group design was adopted to prevent potential crossover effects arising from the long half‐life of reference omalizumab (averaging 24 days in patients with chronic spontaneous urticaria and 26 days in patients with asthma).^3^ For both parts, screening occurred from Day −28 to Day −2, and participants were admitted to the study centre on Day −1 until completion of assessments at 72 h after study drug administration. The first 30 participants were enrolled in part 1 and randomised (1:1) on Day 1 to receive CT‐P39 or EU‐omalizumab. Subsequent participants were enrolled in part 2 and were randomised (1:1:1) on Day 1 to receive CT‐P39, EU‐omalizumab, or US‐omalizumab. The current manuscript describes findings from part 2 of the study.

During part 2 of the study, randomisation codes in block sizes of 6 and 3 were generated by Syneos Health, Inc. using SAS software (SAS Institute Inc.) and provided concealed in envelopes to unblinded pharmacy personnel who dispensed study drugs in sealed cartons for administration. Randomisation was stratified by body weight (<70 kg vs. ≥70 kg), serum total IgE level (<40 international units [IU]/ml vs. ≥40 IU/ml), and sex (male vs. female).

On Day 1, participants received a single dose (150 mg/1 ml) of assigned study drug via a single subcutaneous injection using a PFS into the outer upper arm. Study drugs were administered by designated clinical staff at the study centre who were not blinded to treatment assignment as PFS appearance differed between study drugs. Participants were blinded to treatment assignment through the use of a blindfold, screen, or similar method during study drug administration. Blinded staff who were not present during study drug administration performed all clinical and safety evaluations. All participants were followed up for 127 days (equivalent to approximately five half‐lives of omalizumab), and PK, PD, safety, and immunogenicity were assessed.

This study was conducted in accordance with the Declaration of Helsinki, the International Council for Harmonisation Guideline for Good Clinical Practice, and all applicable laws or regulations. Participants provided written informed consent before enrolment. The informed consent form and study protocol were approved by the Bellberry Human Research Ethics Committee before study initiation.

### 2.2 Participants

Full inclusion and exclusion criteria are included in the Supplementary Methods. Eligible individuals were adults aged 18–55 years (inclusive), who were healthy (defined as having no clinically relevant findings identified by detailed medical history, physical examination, and clinical laboratory tests), weighed >40 and ≤90 kg, with a body mass index of 18–32 kg/m^2^ (inclusive). Eligible individuals had a total IgE level ≤100 IU/ml at screening, to avoid potentially high variability in the PK profile of omalizumab. Key exclusion criteria were: current presence of clinically significant allergic disease, including asthma, urticaria, and eczematous dermatitis; history of anaphylactic shock or hypersensitivity; history of allergic reaction or sensitivity to latex or latex‐derived products; history of and/or concomitant immune complex disease (including type III hypersensitivity), hyperimmunoglobulin E syndrome, autoimmune disease, or bronchopulmonary aspergillosis; current parasitic infection or colonisation on stool evaluation for ova and parasites (assessed in participants considered to be at risk for parasitic infection); history of or any concomitant active malignancy (except adequately treated squamous or basal cell carcinoma of the skin); history of treatment with monoclonal antibodies or other proteins targeting IgE; and pregnancy.

### 2.3 Study endpoints

The primary objective (which was assessed in part 2 of the study) was to evaluate the PK equivalence of CT‐P39 to EU‐omalizumab and US‐omalizumab, assessed in terms of area under the concentration–time curve from time zero to the last quantifiable concentration (AUC_0–last_), area under the concentration–time curve from time zero to infinity (AUC_0–inf_), and maximum serum concentration (*C*_max_). Additional PK parameters were evaluated as secondary endpoints, including the time to maximum serum concentration (*T*_max_), terminal half‐life (*t*_1/2_), terminal elimination rate constant (*λ*_*z*_), apparent total body clearance (CL/F), apparent volume of distribution during the terminal phase (*V*_*z*_/*F*), and percentage of the AUC_0–inf_ obtained by extrapolation (%AUC_ext_). Secondary PD endpoints assessed for free IgE comprised the minimum serum concentration (*C*_min_), time to minimum serum concentration (*T*_min_), and maximum percentage decrease from baseline; for total IgE, they comprised *C*_max_, *T*_max_, and maximum percentage increase from baseline. Safety and immunogenicity were assessed throughout the study.

### 2.4 Study assessments

Blood samples for PK and PD assessments were collected at screening (Day −28 to Day −2; for PD assessment of total IgE only), pre‐dose on Day 1, at 6, 12, 24, 48, and 72 h after the start of study drug administration, and on Days 6, 8, 11, 15, 22, 29, 43, 57, 71, 85, 106, and 127. Validated electrochemiluminescence methods were used to measure total serum omalizumab levels and serum free IgE levels; total IgE (i.e. free and omalizumab‐bound IgE) was measured using ImmunoCAP™ Total IgE (ThermoFisher Scientific). PK parameters were calculated based on measured total serum omalizumab concentrations by non‐compartmental methods, using Phoenix WinNonlin™, version 8.0 (Certara, Inc.).

Safety assessments included monitoring adverse events and prior/concomitant medications throughout. Clinical laboratory assessments (i.e., haematology, clinical chemistry, and urinalysis) were conducted during screening and at Days −1, 3, 8, 15, 29, 57, 85, and 127. Adverse events of special interest (AESIs) were type I allergic reactions (local or systemic, including anaphylaxis and anaphylactic shock), injection‐site reactions (ISRs), type III hypersensitivity (serum sickness/serum sickness‐like reactions, including but not limited to arthritis, arthralgia, rash, fever, or lymphadenopathy, with onset 1–5 days after study drug administration), and helminth infections (including but not limited to cestode, nematode, and trematode infections). Serum samples for immunogenicity assessment were collected pre‐dose on Day 1 and at Days 15, 43, 85, and 127. Anti‐drug antibodies (ADAs) and neutralising antibodies (NAbs; in confirmed ADA‐positive samples) were assessed using electrochemiluminescence assays.

### 2.5 Statistical analysis

Log‐transformed primary endpoints were analysed by analysis of covariance at an alpha level of 0.05. Treatment was a fixed effect and the stratification criteria for randomisation were covariates. PK equivalence was confirmed for each pairwise comparison (CT‐P39 vs. EU‐omalizumab, CT‐P39 vs. US‐omalizumab, and EU‐omalizumab vs. US‐omalizumab) if the 90% confidence intervals (CIs) of the ratios of geometric least‐squares means (gLSMs) were contained within the predefined equivalence margin of 80%–125%. In part 2, a sample size of 42 in each group was required to provide 90% statistical power to demonstrate PK equivalence, based on the equivalence margin, two one‐sided tests, each with an alpha level of 0.05, a 30% coefficient of variation, and an expected ratio of 1.03. Assuming a 15% dropout rate, 147 participants (49 per group) were required for enrolment.

Analysis sets are described in the Supplementary Methods. For baseline characteristics, PD, safety, and additional PK analyses, categorical variables were presented as number of participants (*n*) and percentages; continuous variables were presented using descriptive statistics. Statistical analyses were performed using SAS version 9.4.

## 3 RESULTS

### 3.1 Participant disposition

The first participant was randomised to part 2 of the study on 24 June 2020; the last participant's last visit was on 27 March 2021. In part 2, 146 participants were randomised and received study drug: CT‐P39, *N* = 47; EU‐omalizumab, *N* = 49; US‐omalizumab, *N* = 50 (Supplementary Figure S1). A total of 4 (2.7%) participants withdrew after study drug administration: two withdrew consent (CT‐P39, *n* = 1; EU‐omalizumab, *n* = 1), one was lost to follow‐up (US‐omalizumab group), and one withdrew due to relocating (EU‐omalizumab group). No major protocol deviations leading to exclusion from analysis were reported during the study.

Demographics and baseline characteristics were generally similar between treatment groups (Table 1). Overall, 85 (58.2%) participants were female and 114 (78.1%) were White. At baseline, mean (standard deviation) body weight and serum total IgE were 68.6 (10.6) kg and 34.2 (29.0) IU/ml, respectively, overall.

**TABLE 1:** Participant demographics and baseline characteristics (ITT set—part 2)

	CT‐P39 (*N* = 47)	EU‐omalizumab (*N* = 49)	US‐omalizumab (*N* = 50)
Age (years), median (range)	26.0 (18–55)	29.0 (18–53)	26.5 (18–52)
Sex, *n* (%)^a^			
Female	28 (59.6)	27 (55.1)	30 (60.0)
Male	19 (40.4)	22 (44.9)	20 (40.0)
Race, *n* (%)			
White	36 (76.6)	38 (77.6)	40 (80.0)
Asian	7 (14.9)	6 (12.2)	8 (16.0)
Black or African American	0	3 (6.1)	0
Aboriginal Australian/Torres Strait Islander	1 (2.1)	0	0
Pacific Islander	1 (2.1)	0	0
Other	2 (4.3)	2 (4.1)	2 (4.0)
Weight (kg) on Day −1, mean (SD)	69.3 (11.2)	67.6 (11.1)	68.9 (9.7)
Day −1 weight category, *n* (%)^a^			
<70 kg	26 (55.3)	27 (55.1)	27 (54.0)
≥70 kg	21 (44.7)	22 (44.9)	23 (46.0)
Height (cm) at screening, mean (SD)	169.7 (8.4)	170.1 (9.4)	169.8 (8.8)
BMI (kg/m^2^) at screening, mean (SD)	23.9 (3.0)	23.2 (2.9)	23.7 (3.0)
Serum total IgE at screening (IU/ml), mean (SD)	38.3 (32.3)	30.3 (22.4)	34.0 (31.3)
Serum total IgE category at screening, *n* (%)^a^			
<40 IU/ml	28 (59.6)	33 (67.3)	33 (66.0)
≥40 IU/ml	19 (40.4)	16 (32.7)	17 (34.0)

Abbreviations: BMI, body mass index; EU‐omalizumab, European Union‐approved reference omalizumab; IgE, immunoglobulin E; IU, international units; ITT, intention‐to‐treat; SD, standard deviation; US‐omalizumab, United States‐licensed reference omalizumab.

^a^ Stratification factor.

### 3.2 PK results

**FIGURE 1:**
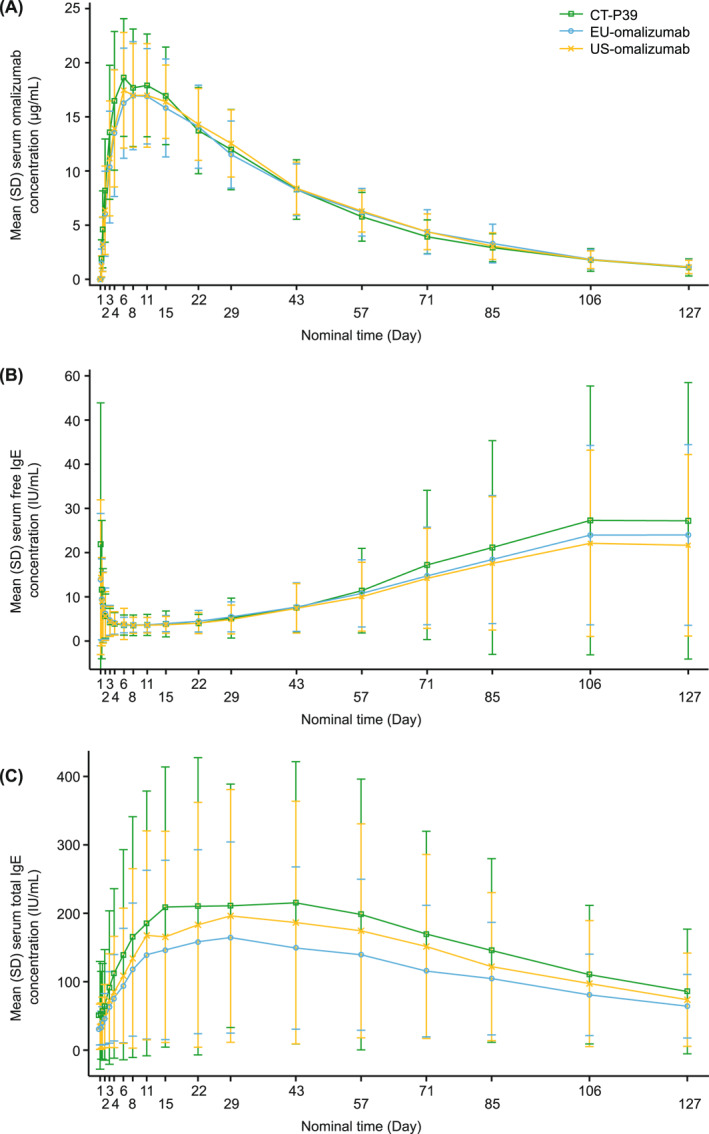
Concentration curves for PK and PD outcomes. (A) Mean (SD) serum concentrations of CT‐P39, EU‐omalizumab, and US‐omalizumab (PK set—part 2). (B) Mean (SD) serum concentrations of free IgE by treatment group (PD set—part 2). (C) Mean (SD) serum concentrations of total IgE by treatment group (PD set—part 2). EU‐omalizumab, European Union‐approved reference omalizumab; IgE, immunoglobulin E; PD, pharmacodynamic; PK, pharmacokinetic; SD, standard deviation; US‐omalizumab, United States‐licensed reference omalizumab

Equivalence was demonstrated between CT‐P39, EU‐omalizumab, and US‐omalizumab in terms of the log‐transformed primary PK endpoints AUC_0–last_, AUC_0–inf_, and *C*_max_ (Table 2). The 90% CIs for the ratios of gLSMs were within the predefined 80%–125% equivalence margin for each pairwise treatment comparison. Primary and secondary PK parameters were generally similar between treatment groups (Table 3), and, following a single administration of study drug, serum omalizumab concentrations were comparable between groups up to Day 127 (Figure 1A).

**TABLE 2:** Statistical analysis^a^ of the primary PK endpoints (PK set—part 2)

Treatment or comparison	*N*	AUC_0–last_ (d·µg/ml) gLSM	AUC_0–inf_ (d·µg/ml) gLSM	*C*_max_ (µg/ml) gLSM
CT‐P39	47	832.06	897.81^b^	19.73
EU‐omalizumab	49	800.07	850.05	17.44
US‐omalizumab	50	837.92	909.42	18.99
Ratio of gLSMs (90% CI)				
CT‐P39 versus EU‐omalizumab		104.00 (94.96–113.89)	105.62 (95.91–116.31)	113.14 (103.15–124.11)
CT‐P39 versus US‐omalizumab		99.30 (90.79–108.61)	98.72 (89.76–108.58)	103.88 (94.83–113.80)
EU‐omalizumab versus US‐omalizumab		95.48 (87.36–104.37)	93.47 (85.09–102.68)	91.82 (83.87–100.52)

Abbreviations: ANCOVA, analysis of covariance; AUC_0–inf_, area under the concentration–time curve from time zero to infinity; AUC_0–last_, area under the concentration–time curve from time zero to the last measurable concentration; CI, confidence interval; *C*_max_, maximum serum concentration; d, day; EU‐omalizumab, European Union‐approved reference omalizumab; gLSM, geometric least‐squares mean; IgE, immunoglobulin E; PK, pharmacokinetic; US‐omalizumab, United States‐licensed reference omalizumab.

^a^ An ANCOVA was performed with the natural log‐transformed PK parameters as the dependent variable, treatment as a fixed effect, and baseline body weight, total IgE level, and sex as covariates.

^b^ Data were analysed for *n* = 46 participants.

**TABLE 3:** Summary of PK parameters (PK set—part 2)

	CT‐P39 (*N* = 47)	EU‐omalizumab (*N* = 49)	US‐omalizumab (*N* = 50)
AUC_0–last_ (d·µg/ml),^a^ mean (SD)	846.0 (251.9)	843.8 (248.0)	850.0 (213.1)
AUC_0–inf_ (d·µg/ml),^a^ mean (SD)	910.9 (278.6)^b^	897.7 (270.3)	926.3 (273.3)
*C*_max_ (µg/ml),^a^ mean (SD)	20.08 (5.994)	18.24 (4.998)	19.43 (5.416)
*T*_max_ (d), median (range)	7.098 (2.00–14.26)	7.306 (3.00–18.26)	7.183 (3.00–21.17)
*t*_1/2_ (d), mean (SD)	29.30 (9.516)^b^	27.69 (5.560)	28.63 (6.629)
*λ*_*z*_ (1/d), mean (SD)	0.02543 (0.006227)^b^	0.02619 (0.006173)	0.02536 (0.005447)
CL/F (L/d), mean (SD)	0.1820 (0.06204)^b^	0.1850 (0.07120)	0.1764 (0.05601)
*V*_*z*_/*F* (L), mean (SD)	7.258 (1.912)^b^	7.172 (2.176)	7.011 (1.808)
%AUC_ext_ (%), mean (SD)	5.513 (5.429)^b^	5.669 (3.783)	6.519 (9.642)

Abbreviations: %AUC_ext_, percentage of the AUC_0–inf_ obtained by extrapolation; AUC_0–inf_, area under the concentration–time curve from time zero to infinity; AUC_0–last_, area under the concentration–time curve from time zero to the last measurable concentration; CL/F, apparent total body clearance; *C*_max_, maximum serum concentration; d, day; EU‐omalizumab, European Union‐approved reference omalizumab; PK, pharmacokinetic; SD, standard deviation; *t*_1/2_, terminal half‐life; *T*_max_, time to maximum concentration; US‐omalizumab, United States‐licensed reference omalizumab; *V*_*z*_/*F*, apparent volume of distribution during terminal phase; *λ*_*z*_, terminal elimination rate constant.

^a^ Primary PK parameter.

^b^ Data were analysed for *n* = 46 participants.

### 3.3 PD results

Mean serum concentrations of free IgE rapidly decreased following study drug administration, to a comparable degree for each of the three treatment groups (Figure 1B). Serum free IgE levels increased towards baseline at the end of the study. Mean serum total IgE concentrations were elevated following study drug administration, to comparable levels between treatment groups, and decreased towards baseline at the end of the study (Figure 1C).

PD parameters for free and total IgE were generally comparable between treatment groups (Table 4). Although there were some numerical differences in maximum percentage increase in total IgE, these were likely accounted for by the high inter‐participant variability. There was one notable outlier in the US‐omalizumab group, with a maximum increase exceeding 10,000% (data not shown).

**TABLE 4:** Summary of PD parameters^a^ (PD set—part 2)

	CT‐P39 (*N* = 46)	EU‐omalizumab (*N* = 49)	US‐omalizumab (*N* = 49)
Free IgE			
*C*_min_ (IU/ml)			
*n*	31	38	32
Mean (SD)	3.527 (1.453)	3.559 (1.562)	3.700 (1.696)
*T*_min_ (days)			
*n*	31	38	32
Median (range)	3.000 (0.25–21.21)	5.051 (0.25–70.17)	3.002 (0.25–41.07)
Maximum % decrease (%)			
*n*	30	38	32
Mean (SD)	73.05 (21.25)	64.20 (24.92)	66.62 (24.81)
Total IgE			
*C*_max_ (IU/ml)			
*n*	46	49	49
Mean (SD)	245.2 (223.2)	174.3 (145.2)	219.6 (187.4)
*T*_max_ (day)			
*n*	46	49	49
Median (range)	28.19 (3.00–70.13)	28.16 (14.01–71.07)	28.09 (10.18–73.07)
Maximum % increase (%)			
*n*	45	48	47
Mean (SD)	574.3 (309.4)	494.5 (235.4)	857.8 (2146.9)

Abbreviations: *C*_max_, maximum serum concentration; *C*_min_, minimum serum concentration; EU‐omalizumab, European Union‐approved reference omalizumab; IgE, immunoglobulin E; IU, international units; PD, pharmacodynamic; SD, standard deviation; *T*_max_, time to maximum concentration; *T*_min_, time to minimum concentration; US‐omalizumab, United States‐licensed reference omalizumab.

^a^ PD parameters were not calculated for participants with free IgE concentrations below the lower limit of quantification at baseline. Maximum % decrease and maximum % increase were not calculated for participants with a free IgE concentration above the upper limit of quantification at baseline or a total IgE concentration below the lower limit of quantification at baseline, respectively.

### 3.4 Safety

All participants successfully received a single dose of study drug. Overall, 284 treatment‐emergent adverse events (TEAEs) were reported in 33 (70.2%), 37 (75.5%), and 34 (68.0%) participants in the CT‐P39, EU‐omalizumab, and US‐omalizumab groups, respectively (Table 5). Correspondingly, 22 (46.8%), 30 (61.2%), and 27 (54.0%) participants experienced study drug‐related TEAEs. Most TEAEs were grade 1–2 in intensity. There were only two TEAEs of grade ≥3: head injury caused by assault (*n* = 1; CT‐P39 group) and syncope (*n* = 1; EU‐omalizumab group); both were grade 3 in intensity and evaluated by investigators to be unrelated to the study drug. The head injury was the sole treatment‐emergent serious adverse event (TESAE) reported in this study, and the participant recovered with medical treatment. Headache was the most frequent TEAE, reported by 11 (23.4%), 10 (20.4%), and 15 (30.0%) participants in the CT‐P39, EU‐omalizumab, and US‐omalizumab groups, respectively (Supplementary Table S1). There were no TEAEs leading to study discontinuation or death.

**TABLE 5:** Summary of adverse events (safety set—part 2)

	CT‐P39 (*N* = 47)	EU‐omalizumab (*N* = 49)	US‐omalizumab (*N* = 50)
Total number of TEAEs	79	95	110
Total number of TESAEs	1	0	0
Participants with ≥1 TEAE, *n* (%)	33 (70.2)	37 (75.5)	34 (68.0)
Study drug‐related TEAE	22 (46.8)	30 (61.2)	27 (54.0)
Participants with ≥1 TESAE, *n* (%)	1 (2.1)	0	0
Participants with ≥1 TEAE leading to study discontinuation, *n* (%)	0	0	0
Participants with ≥1 TEAE leading to death, *n* (%)	0	0	0
Participants with ≥1 TEAE of ISR, *n* (%)^a^	8 (17.0)	5 (10.2)	6 (12.0)

Abbreviations: AESI, adverse event of special interest; EU‐omalizumab, European Union‐approved reference omalizumab; ISR, injection‐site reaction; TEAE, treatment‐emergent adverse event; TESAE, treatment‐emergent serious adverse event; US‐omalizumab, United States‐licensed reference omalizumab.

^a^ No other AESIs were reported during the study.

Considering AESIs, there were no TEAEs classified as type I allergic reactions (including anaphylaxis), serum sickness/serum sickness‐like reactions, or helminth infections during the study. ISR was the only AESI reported and the second most frequently reported TEAE overall (Supplementary Table S1). ISRs were reported by 8 (17.0%), 5 (10.2%), and 6 (12.0%) participants in the CT‐P39, EU‐omalizumab, and US‐omalizumab groups, respectively (Table 5). The most frequently reported manifestation of ISR was injection‐site erythema reported in 5 (10.6%), 3 (6.1%), and 3 (6.0%) of participants in the CT‐P39, EU‐omalizumab, and US‐omalizumab groups, respectively. All ISRs were grade 1 in intensity and all participants recovered from the event.

Overall, there were no notable differences between treatment groups in terms of clinical laboratory findings, and no notable safety findings in terms of vital signs, physical findings, or other observations.

Immunogenicity was lower in the CT‐P39 group than in the reference omalizumab groups. After study drug administration, 1 (2.1%), 13 (26.5%), and 18 (36.0%) participants in the CT‐P39, EU‐omalizumab, and US‐omalizumab groups, respectively, had at least one positive ADA result. At least one positive NAb result was reported in 1 (2.0%) and 3 (6.0%) participants in the EU‐omalizumab and US‐omalizumab groups, respectively. No participants in the CT‐P39 group had detectable positive NAb titres.

## 4 DISCUSSION

Mean peak exposure and total exposure are key parameters for the demonstration of PK similarity of a candidate biosimilar to its reference product during a biosimilar clinical development programme.^16^, ^17^, ^21^ The present study showed that single doses of CT‐P39, EU‐omalizumab, and US‐omalizumab were equivalent in terms of PK, according to predefined equivalence criteria. In this study, the 90% CIs for each primary PK endpoint were contained entirely within the predefined 80%–125% equivalence margin. Therefore, equivalence was demonstrated between CT‐P39, EU‐omalizumab, and US‐omalizumab in terms of PK. After study drug administration, free IgE decreased while total IgE increased. PD results for free IgE were comparable between groups in terms of *C*_min_, *T*_min_, and maximum percentage decrease. Increases in total IgE level were comparable between groups, although numerical differences were noted for the maximum percentage increase in total IgE. This was likely driven by inter‐participant variation and/or aberrant values, with one notable outlier observed in the US‐omalizumab group. The value was greatly divergent from that for other participants, but no clear factor that could account for the difference was identified. Secondary PK parameters and safety findings were generally comparable between treatment groups.

In the current study, the PK parameters observed across groups were broadly similar to those previously noted in a study evaluating reference omalizumab in adults with stable atopic disease.^22^ Importantly, reference omalizumab and/or free IgE concentrations have been demonstrated to correlate with clinical outcomes in populations of patients with asthma or urticaria,^23^, ^24^, ^25^ underlining the importance of assessing these measures when evaluating candidate biosimilars of reference omalizumab. Analytical tests have demonstrated that CT‐P39 is similar to EU‐omalizumab and US‐omalizumab, in terms of primary‐ and higher‐order structure, modifications, post‐translational forms, purity/impurity, and biological activity (data not shown). Combined with previous findings, the comparability of PK and PD outcomes between CT‐P39 and reference omalizumab in the current study suggests that CT‐P39 may be expected to have comparable efficacy to the reference product in future evaluations.

Overall, the safety profile of CT‐P39 was comparable to those of EU‐omalizumab and US‐omalizumab. In this study, the most frequently reported TEAE was headache. This is broadly aligned with the ‘common’ incidence of headache described in the EU prescribing information for reference omalizumab, which is based on clinical studies in patients with allergic asthma, chronic rhinosinusitis with nasal polyps, and chronic spontaneous urticaria.^2^ Headache was also the most common TEAE reported during a study of reference omalizumab administered to adult patients with stable atopic disease.^22^ Importantly, there were no TEAEs leading to study discontinuation or death, and only one TESAE occurred (head injury caused by assault), which was not treatment related. Furthermore, of the AESIs evaluated in the study, only ISRs were observed and the frequency of these events was in keeping with the ‘common’ incidence reported in the EU prescribing information for reference omalizumab.^2^

Notably, lower immunogenicity was observed in the CT‐P39 group than in the two reference omalizumab groups. However, these results should be interpreted with caution as the sample size was relatively small. In addition, no participant experienced an increase in ADA titre after treatment and the majority of ADA‐positive participants had a transiently low ADA titre of 1 in 25, the minimum required dilution of the assay. Relatively high immunogenicity was reported for reference omalizumab in this study compared with that in previous studies in patients with allergic disease,^3^, ^26^ possibly due to the use of a more sensitive and drug‐tolerant ADA assay technology. ADA detection is highly dependent on assay sensitivity and specificity, making comparisons between studies difficult.^3^ Nevertheless, NAb detection was low across groups in this study.

A limitation of the present study is that most (>75%) participants in each group were White, potentially limiting generalisability to other racial or ethnic groups. However, previous research with reference omalizumab has not found any clinically meaningful differences in PK and PD based on race, and efficacy and safety profiles do not differ by race or ethnicity.^25^, ^27^, ^28^, ^29^ Building on the findings of the present study, the double‐blind, randomised, parallel‐group, Phase 3 CT‐P39 3.1 study (NCT04426890) is ongoing and aims to demonstrate equivalence of the clinical efficacy, PK, PD, and safety of CT‐P39 versus EU‐omalizumab in patients with chronic spontaneous urticaria.^30^ The primary endpoint of the study will assess therapeutic similarity between CT‐P39 and EU‐omalizumab.^30^

In conclusion, this study demonstrated PK equivalence of CT‐P39 to EU‐omalizumab and US‐omalizumab following administration of a single dose in healthy individuals. As part of a stepwise biosimilar development programme, this study provides PK evidence of high similarity for CT‐P39 and reference omalizumab. A single dose of CT‐P39 was found to be safe and well tolerated.

## AUTHOR CONTRIBUTIONS

All authors have made substantial contributions to the conception and design of the study or the acquisition, analysis, or interpretation of data; drafted the manuscript or revised it critically for important intellectual content; given final approval of the version to be published; and agreed to be accountable for all aspects of the work in ensuring that questions related to the accuracy or integrity of any part of the work are appropriately investigated and resolved.

## CONFLICT OF INTEREST

Marcus Maurer reports current or recent speaker and/or advisory roles for and/or has received research funding from Allakos, Amgen, Aralez, Argenx, AstraZeneca, Celldex, Celltrion, Centogene, CSL Behring, FAES, Genentech, GIInnovation, Innate Pharma, Kyowa Kirin, Leo Pharma, Lilly, Menarini, Moxie, Novartis, Roche, Sanofi/Regeneron, ThirdHarmonicBio, UCB, and Uriach. Sarbjit S. Saini reports research interests for Amgen, National Institutes of Health, Novartis, Regeneron, and Sanofi; and consultancies for Allakos, Aquestive, Celltrion, Escient, Granular Therapeutics, Innate, Novartis, Regeneron, and Sanofi. Sunghyun Kim, Keumyoung Ahn, Suyoung Kim, and Sewon Lee are employees of Celltrion, Inc. Clive Grattan reports consultancies for Argenx, Blueprint Medicines, Celltrion, Inc., Novartis, and Sanofi. Kristi McLendon and Paul Wabnitz have no conflicts of interest to declare.

## PRIOR PRESENTATION

Selected results from the CT‐P39 1.1 study have been presented as posters at the Urticaria Centers of Reference and Excellence (UCARE) meeting (Hiroshima, Japan; 9–11 December 2021) and the American Academy of Allergy Asthma & Immunology (AAAAI) Annual Meeting (Phoenix, AZ, USA; 25–28 February 2022).

## Supporting information

Supporting Information S1
