# Supplementary material for: Pharmacokinetic equivalence of CT‐P39 and reference omalizumab in healthy individuals: A randomised, double‐blind, parallel‐group, Phase 1 trial
Source: Clin Transl Allergy. 2022 Nov 15;12(11):e12204. doi: 10.1002/clt2.12204 (PMC9665328; doi:10.1002/clt2.12204)
Supplement: Supplementary file 1 — Supporting Information S1 [file CLT2-12-e12204-s001.pdf]

Pharmacokinetic equivalence of CT-P39 and reference omalizumab in healthy individuals: a randomised, double-blind, parallel-group, Phase 1 trial

Marcus Maurer, Sarbjit S. Saini, Kristi McLendon, Paul Wabnitz, Sunghyun Kim, Keumyoung Ahn, Suyoung Kim, Sewon Lee, Clive Grattan

Correspondence to: Dr Clive Grattan, Specialist Ambulatory Directorate, St John's Institute of Dermatology, Guy's Hospital, Great Maze Pond Road, London, SE1 9RT, UK

Email: Clive.E.Grattan@gstt.nhs.uk

Supporting information

Supplementary methods

Full inclusion and exclusion criteria

Inclusion criteria

1. Healthy males or females aged 18–55 years (healthy defined as no clinically relevant abnormalities identified by a detailed medical history, full physical examination [including blood pressure, heart rate, and 12-lead electrocardiogram], and clinical laboratory tests prior to study drug administration).
2. Body weight >40 kg and ≤90 kg, and body mass index of 18–32 kg/m^2.
3. Total immunoglobulin E (IgE) level ≤100 IU/mL at screening.
4. Able to understand and comply with protocol requirements, instructions, and restrictions.
5. Voluntarily agreed to participate in the study and provided written informed consent prior to undergoing any of the screening procedures.
6. Individuals and their partners of childbearing potential to agree to use a highly effective method of contraception.

Exclusion criteria

1. Current presence of allergic state, such as asthma, urticaria, angioedema, and eczematous dermatitis, considered as clinically significant by the investigator.
2. History of anaphylactic shock or hypersensitivity, including known or suspected clinically relevant hypersensitivity to any components of the study drugs or other similar drugs (e.g., monoclonal antibodies and human intravenous immunoglobulin).

3. History of allergic reactions or sensitivity to latex or latex-derived products.
4. History of and/or concomitant immune complex disease (including type III hypersensitivity), hyperimmunoglobulin E syndrome, autoimmune disease, or bronchopulmonary aspergillosis.
5. Current parasitic infection or colonisation on stool evaluation for ova and parasites in those with the following risk factors for parasitic disease: travel within 6 months to, or living in, an endemic area; chronic gastrointestinal symptoms; chronic immunosuppression; or absolute eosinophil count more than two times the upper limit of normal.
6. History of and/or current medical condition, including cardiac, gastrointestinal, renal, hepatic, haematological (including pancytopenia, aplastic anaemia, or blood dyscrasia), metabolic (including known diabetes mellitus), neurologic, or pulmonary diseases, or psychiatric condition classed as clinically significant by the investigator.
7. History of or any concomitant active malignancy, except adequately treated squamous or basal cell carcinoma of the skin.
8. A known infection with human immunodeficiency virus, hepatitis B, or hepatitis C, or any active infection requiring treatments, except adequately treated and completely recovered past infections.
9. History of and/or current illness within 28 days prior to the study drug administration that was identified as clinically significant by the investigator.
10. History of surgical intervention or an operation within 28 days prior to the study drug administration, or surgical procedure planned during the study period.
11. History of and/or concurrent use of prescription medications (excluding hormonal birth control), over-the-counter drugs, dietary supplements, or herbal remedies from 7 days or 5 half-lives (whichever is longer) prior to study drug administration until completion of the study.

12. Treatment with an investigational drug, any monoclonal antibody, or fusion protein; current use of biologics; or participation in another clinical trial within 3 months or 5 half-lives (whichever is longer) prior to study drug administration.
13. History of and/or concurrent treatment with an anti-IgE monoclonal antibody or any other antibody or protein targeting IgE.
14. Pregnant, lactating, or planning to be pregnant or breastfeed during the study period; or planning to father a child or donate sperm during the study period.
15. Reasonable evidence or history of drug, alcohol, or nicotine abuse prior to study drug administration, defined as: positive result for drug urine test during screening or at Day −1; history or presence of regular consumption exceeding an average weekly intake of >14 units of alcohol in the 3 months prior to study drug administration (a standard unit is equal to approximately 285 mL of full strength beer [4.8% alcohol by volume; ABV], 30 mL of spirits [40% ABV], or 100 mL of wine [13.5% ABV]); or consumption of >10 cigarettes or equivalent per day within 28 days prior to study drug administration.
16. Unwilling to avoid the use of alcohol or alcohol-containing foods, medications, or beverages within 24 hours prior to each study visit and/or inability to refrain from smoking during in-house stays.
17. Whole blood donation or blood loss of ≥450 mL within 8 weeks (plasma/platelet donation within 4 weeks) prior to study drug administration.
18. Evidence of a condition (including psychological disorder, emotional problems, or resultant therapy) that was likely to invalidate informed consent or confer a limited ability to comply with the protocol requirements in the opinion of the investigator.
19. Vulnerable individuals (e.g., employees of the clinical trial site or any other individuals involved with the conduct of the study or immediate family members of such individuals, persons kept in prison, or other persons institutionalised by law enforcement).

20. Presence of tattoos, sunburn, or other skin disturbances (including cuts, bruises, redness, hardness, or tenderness) on both the left and right upper arm that might interfere with a medical assessment of the injection site both prior to and following study drug administration.

21. Individuals not likely to complete the study for whatever reason in the opinion of the investigator.

Analysis sets

Pharmacokinetic (PK), pharmacodynamic (PD), and safety analyses were conducted in the PK, PD, and safety sets, respectively, while the intention-to-treat (ITT) set was used for analysis of participant demographics and baseline characteristics. Analysis sets were as defined below:

ITT set: all participants enrolled and randomly assigned to receive a dose of study drug (regardless of whether any study drug was administered).

PK set: all participants who received a complete dose of study drug and who had ≥1 post-treatment PK result with a concentration above the lower limit of quantification for omalizumab.

PD set: all participants who received a complete dose of study drug and who had ≥1 post-treatment free IgE or total IgE concentration above the lower limit of quantification.

Safety set: all randomly assigned participants who received a full or partial dose of study drug.

Supplementary results

Supplementary Figure S1. Participant disposition.

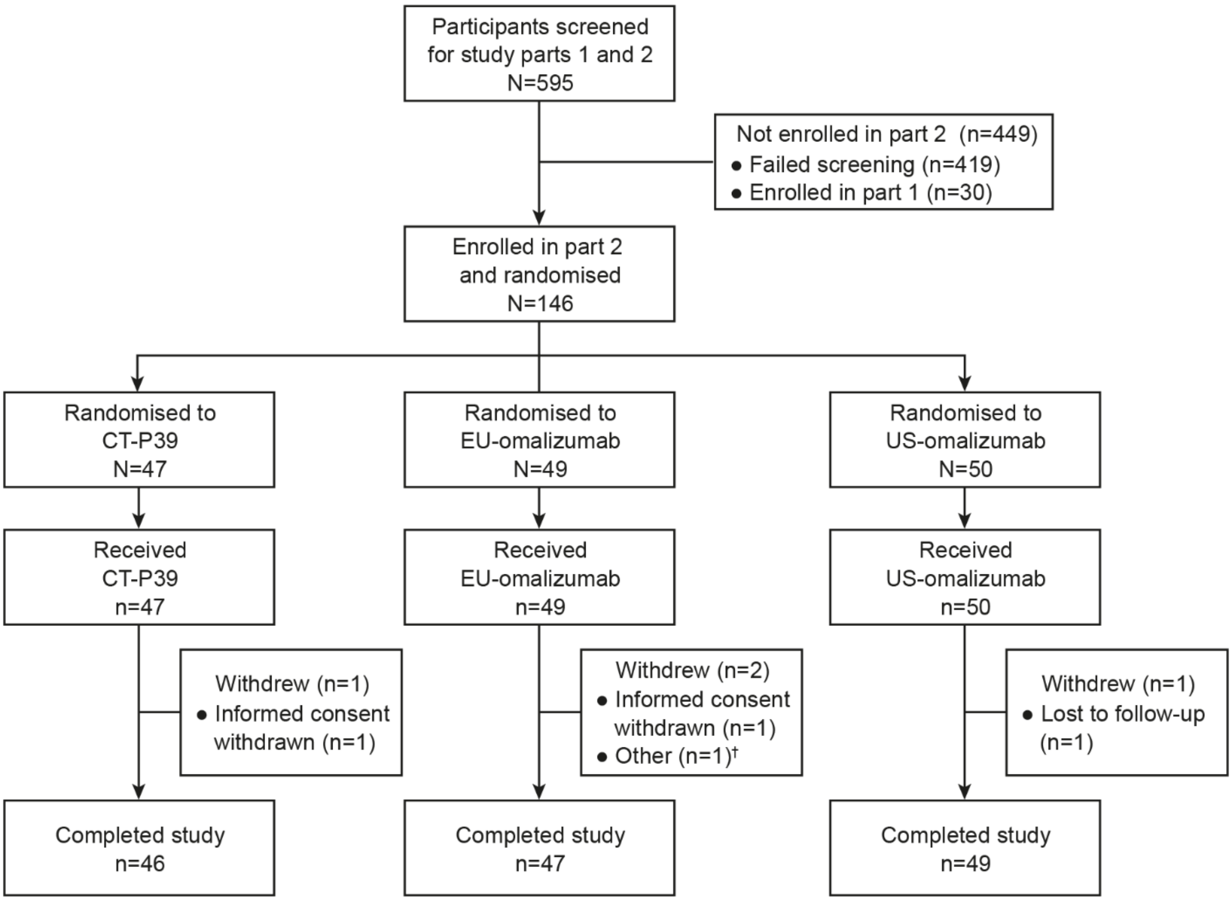

†Participant withdrew as they relocated.

EU-omalizumab, European Union-approved reference omalizumab; US-omalizumab, United States-licensed omalizumab.

Supplementary Table S1. TEAEs experienced by ≥5% of participants in any group by System Organ Class and Preferred Term (safety set – part 2).

**System Organ Class, Preferred Term, n (%)**	**CT-P39 (N=47)**	**EU-omalizumab (N=49)**	**US-omalizumab (N=50)**
Gastrointestinal disorders			
Diarrhoea	0	0	4 (8.0)
Nausea	6 (12.8)	2 (4.1)	2 (4.0)
Vomiting	3 (6.4)	0	0
General disorders and administration-site conditions			
Catheter-site irritation	1 (2.1)	1 (2.0)	3 (6.0)
Catheter-site pain	0	1 (2.0)	3 (6.0)
Fatigue	3 (6.4)	2 (4.1)	3 (6.0)
Injection-site reaction	8 (17.0)	5 (10.2)	6 (12.0)
Infections and infestations			
Rhinitis	0	4 (8.2)	2 (4.0)
URTI	3 (6.4)	1 (2.0)	0
Injury, poisoning, and procedural complications			
Contusion	3 (6.4)	0	2 (4.0)
Musculoskeletal and connective tissue disorders			
Back pain	0	2 (4.1)	4 (8.0)
Myalgia	1 (2.1)	1 (2.0)	4 (8.0)
Nervous system disorders			
Dizziness	1 (2.1)	4 (8.2)	2 (4.0)
Headache	11 (23.4)	10 (20.4)	15 (30.0)
Skin and subcutaneous tissue disorders			
Rash	1 (2.1)	1 (2.0)	3 (6.0)

TEAE, treatment-emergent adverse event; URTI, upper respiratory tract infection.
